# Barriers and facilitators to pharmacotherapy for alcohol use disorders in primary care: results of a qualitative study in 4 va primary care clinics

**DOI:** 10.1186/1940-0640-10-S2-P12

**Published:** 2015-09-24

**Authors:** Emily Williams, CE Achtmeyer, JP Young, KA Bradley, D Berger, MB Siegel, G Curran, EJ Ludman, GT Lapham, AHS Harris, M Forehand

**Affiliations:** 1 Center of Innovation for Veteran-Centered Value-Driven Care, Health Services Research & Development, Veterans Affairs Puget Sound Health Care System, Seattle, USA; 2 Department of Health Services, School of Public Health, University of Washington, Seattle, USA; 3 Group Health Research Instistute, Seattle, USA; 4 Department of Medicine, School of Medicine, University of Washington, Seattle, USA; 5 Primary and Specialty Care Medicine, Veterans Affiars Puget Sound Health Care System, Seattle, USA; 6 Department of Social and Behavioral Sciences, School of Public Health, Boston University, Boston, USA; 7 Division of Health Services Research, Department of Psychiatry, University of Arkansas for Medical Sciences, Arkansas, USA; 8 Palo Alto Health Care System, Veterans Health Administration, Palo Alto, USA; 9 Foster School of Business, University of Washington, Seattle, USA

## Background

In the context of routine population-based alcohol screening to identify primary care (PC) patients who might benefit from brief intervention, many patients identified will have alcohol use disorders (AUD)[1] and will likely require more intensive treatments. FDA approved medications are recommended to treat AUD[2]^b^ and could be offered in PC. Currently, use of AUD medications is extremely rare.[3] This qualitative study sought to understand barriers and facilitators to prescribing AUD medications in PC.

## Material and methods

Key contacts and snowball sampling were used to recruit 23 PC providers (MDs and NPs) from 4 Veterans Health Administration (VA) clinics. Providers completed semi-structured interviews in person, which were recorded, transcribed, and analyzed using rapid team-based qualitative methods.

## Results

Few participating providers had prescribed AUD medications. Providers consistently reported concern regarding lack of time to adequately address AUD, need for training in prescribing AUD medications, and need for ongoing support from mental health or on-site staff to provide behavioral counseling. However, some participating providers were more willing than others to consider prescribing AUD medications. Providers who were more willing viewed prescribing for AUD as part of their role as a PC provider, framed medications as a potentially effective “tool” or “foot in the door” for treating AUD, and believed that providing AUD medications in PC may catalyze change while reducing stigma and other barriers to specialty addictions treatment. Those who were less willing believed that substantial programmatic changes would be needed to facilitate provision of AUD medications in PC, had less belief in the ability of “pills” to treat AUD, and believed AUD treatment was best left to specialty settings.

## Conclusions

With training and additional behavioral staff, it may be possible to capitalize on some providers' willingness and optimism to increase provision of medications as part of PC for patients with AUD.
